# Detection of suitable habitat areas in Japan of the Lyme disease and tick-borne encephalitis vectors *Ixodes ovatus* and *Ixodes persulcatus* based on abiotic factors

**DOI:** 10.1016/j.crpvbd.2026.100385

**Published:** 2026-05-14

**Authors:** Patrick H. Kelly, Harrison M. Marick, Julie Davis, Ai Takano, Kentaro Yoshii, Hiroki Kawabata, Kentaro Itokawa, Kozue Sato, Alice C.C. Lau, Yongjin Qiu, Andreas Pilz, Agustín Estrada-Peña, Frederick J. Angulo, Shuhei Ito, Jennifer C. Moïsi, Yoshikazu Nakayama

**Affiliations:** a U.S. Medical Affairs, Vaccines, U.S. Commercial Division, Pfizer, Collegeville, PA, USA; b Tiber Solutions, VA, USA; c Global Vaccines Medical Affairs, Pfizer Research & Development, Boston, MA, USA; d Department of Veterinary Epidemiology, Joint Faculty of Veterinary Medicine, Yamaguchi University, Yamaguchi, Japan; e National Research Center for the Control and Prevention of Infectious Diseases (CCPID), Nagasaki University, Nagasaki, Japan; f National Institute of Infectious Diseases (NIID), Tokyo, Japan; g Global Vaccines Medical Affairs, Pfizer Research & Development, Pfizer, Vienna, Austria; h Senior External Consultant, Ministry of Health, Government of Spain, Madrid, Spain; i Vaccines Medical Affairs, Pfizer Japan, Tokyo, Japan; j Global Vaccines Medical Affairs, Pfizer Research & Development, Paris, France

**Keywords:** Lyme disease, TBE, Tick;, *Ixodes*, Ixodid, *Borrelia burgdorferi*, Japan

## Abstract

In Japan, Lyme disease and tick-borne encephalitis (TBE) are primarily in the northernmost prefecture Hokkaido, where their primary vectors *Ixodes ovatus* and *Ixodes persulcatus* are most abundant. Recently, tick surveillance activities have collected both species across Japan, indicating potential expansion of their tick-borne pathogens (TBPs). We built a machine-learning (ML) model using available tick surveillance and environmental data to predict suitable habitat areas for *I. ovatus* and *I. persulcatus* and identify potential higher-risk areas of Lyme disease and TBE across Japan. Data on the occurrence and abundance of 11 vector tick species between 1990 and 2023 were extracted from studies identified *via* systematic literature search in two online databases or provided by local experts for ML development. After multiple iterations and permutations, separate Random Forest ML algorithms for *I. ovatus* and *I. persulcatus* were trained *via* 26 abiotic variables of climate and topography based on the occurrence and abundance respective to each species. Data on 93,289 ticks from 57 sources were extracted, and the ML algorithms’ area under the curves were high (> 0.89). Climate-related variables were the strongest predictors (> 90% cumulative model importance) for both *I. ovatus* and *I. persulcatus*. High suitability for both species was identified in Hokkaido and cooler, wetter regions in central Honshu, while *I*. *ovatus* had a broader ecological niche than *I. persulcatus*, with moderate suitability in mountainous regions of central Kyushu and surrounding the Tokyo Bay area. Our ML models suggest high suitability areas for *Ixodes* vectors may be widespread in Japan, indicating expanding potential risks of TBPs.

## Highlights

• A dataset with 93,289 ticks on 11 species in Japan was compiled and provided.
• Suitable areas for *Ixodes* vectors are predicted throughout Japan.
• Tick-borne diseases by *Ixodes* ticks may be more widespread in Japan.
• Machine-learning is a useful approach to inform potential tick-borne risks.

## Graphical abstract

**Figure:**
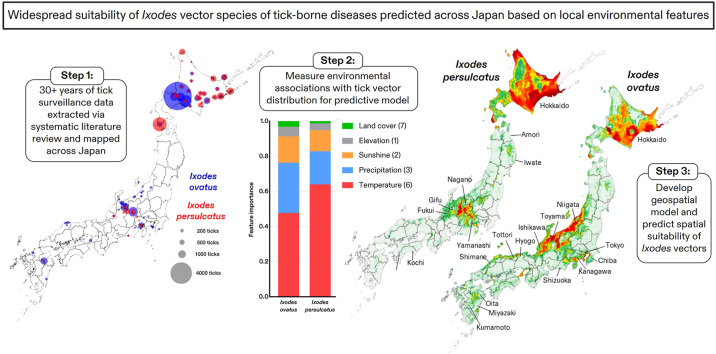

## 1 Introduction

*Ixodes* ticks are the primary vectors of several etiological agents of significant public health threats, including Lyme disease (Eisen, 2020), tick-borne encephalitis (TBE) (Dobler et al., 2011), babesiosis (Zamoto-Niikura et al., 2016), anaplasmosis (Bakken and Dumler, 2008), and relapsing-fever (Krause et al., 2015). In Japan, multiple hard tick (Ixodidae) competent vector species of human disease-causing pathogens are endemic (Yamaji et al., 2018), including the primary vectors of the etiological agents of Lyme disease and TBE, *Ixodes ovatus* and *Ixodes persulcatus*. Across Japan, *I. ovatus* and *I. persulcatus* are most common in the northernmost prefecture of Hokkaido, where the majority of human cases of Lyme disease and TBE have been reported (Miyamoto et al., 1992; Yanagihara and Masuzawa, 1997; NIID, 2025). In part due to increased public awareness of tick-borne diseases in Japan caused by the emergence of severe fever with thrombocytopenia syndrome (SFTS), the number of nationally reported annual cases of Lyme disease has gradually increased, with estimated areas of infection expanding outside of Hokkaido since 2014 (NIID, 2025). The first human cases of TBE were also recently observed among patients residing in Oita, Tokyo, and Okayama with no reported travel-histories (Ohira et al., 2023), underscoring the potential for increased risks of Lyme disease and TBE to populations outside of Hokkaido.

The interplay across features of climate, habitat and availability of animal hosts and reservoirs drives the enzootic risks of *Ixodes* vectors and their tick-borne pathogens (Humair and Gern, 1998; Moustafa et al., 2016; Sato et al., 2021; Doi et al., 2018; Ito et al., 2024a). *Ixodes* ticks are generalist feeders with low host-preferential specificity feeding on a wide spectrum of mammalian hosts capable of serving as competent reservoirs of tick-borne pathogens that cause human disease (Kahl and Gray, 2023). Compared to other *Ixodes* tick vectors of Lyme disease and TBE in the northern hemisphere, *I*. *ovatus* and *I. persulcatus* have shown greater resilience to cold and dry environments at higher altitudes, though each species exhibits different geographical distributions and habitat suitability across the diverse ecologies throughout Japan (Doi et al., 2021a; Ito et al., 2024b; Shimizu et al., 2024). *Ixodes ovatus* is associated with a variety of wildlife hosts and widely distributed across regions of Japan, increasing the likelihood of enzootic transmission of *Borrelia burgdorferi* (*sensu lato*) and TBE virus (TBEV), the causative agents of Lyme disease and TBE, respectively, to humans (Ishiguro et al., 1995; Yoshii, 2017). Concurrently with increasing temperatures continuing to rise in Japan, changing land-use patterns from habitat fragmentation and fewer farming and hunting communities have contributed to the emergence of novel tick-borne pathogens like Yezo virus (Kumar et al., 2024) by increasing the abundance and suitable habitats for important tick hosts and reservoirs and allowing for the geographical expansion of *I. ovatus* and *I. persulcatus* (Doi et al., 2018; Yamaji et al., 2018). Historically, both *I. ovatus* and *I. persulcatus* have been collected in Hokkaido, Prefectures in western (e.g. Niigata and Fukui) and central (Gifu, Nagano, and Fukushima) Honshu, and southern regions in the higher altitudes (> 1700 m) of Shikoku (Ehime and Kochi) and Kyushu (Oita), with changing abundances reported over time (Takada, 1990; Ishiguro et al., 1992; Miyamoto et al., 1992; Takada et al., 1994; Nakao et al., 1996; Hashimoto et al., 2002; Sato et al., 2021; Ikeda et al., 2025). Recent tick surveillance and multiple mammalian serological studies have also recorded the geographical spread of *Ixodes* spp. and Lyme disease borreliae and TBEV within and outside of Hokkaido (Doi et al., 2021a, 2021b, b; Zamoto-Niikura et al., 2023; Ito et al., 2024a, 2024b). Therefore, it is imperative to assess the potential endemicity of Lyme disease and TBE in other areas of Japan and better inform populations at risk of tick-borne diseases.

Predictive modeling and machine-learning (ML) algorithms are powerful tools for supporting public health activities on tick-borne diseases by integrating epidemiological surveillance with geospatial environmental data to project potential distributions of vector tick species and their associated pathogens (Burtis et al., 2022; Kelly et al., 2025; Dagostin et al., 2026). Several studies have developed predictive models to determine potential risk areas of tick-borne diseases in Japan (Matsuyama et al., 2020; Ogawa et al., 2024), but relatively few have investigated *I. ovatus* or *I. persulcatus* and their associated pathogens or utilized ML approaches (Doi et al., 2021a; Ito et al., 2024b). This study sought to develop an ML algorithm using historical tick surveillance data and publicly available abiotic variables to provide spatial projections of the suitable habitat areas for *I. ovatus* and *I. persulcatus* across Japan. Two main goals in the scope of this study were to: (i) determine potential new geographies for each species and compare their varying spatial niches; and (ii) advance our understanding of which prefectures and populations may be at increased risk of Lyme disease and TBE. Ultimately, we aimed to support the identification of potential human exposure risks to infected *Ixodes* ticks to improve public health outcomes against the rising threat of tick-borne diseases in Japan.

## 2 Materials and methods

### 2.1 Literature review and data retrieval

A systematic literature search guided by the Preferred Reporting Items for Systematic Review and Meta-Analyses (Page et al., 2021) was conducted in PubMed and Web of Science databases between 1 September 2023 and 7 February 2025 to identify publicly available studies with relevant tick surveillance data for ML algorithm development. Tick surveillance studies in Japan published after 1 January 1990 were retrieved using the following template string of key terms and associated derivatives in the English and Japanese languages: (“Japan”[tiab]) AND (“Ixodes”[tiab] OR “Ixodid”[tiab] OR “tick”[tiab] OR “ticks”[tiab] OR “persulcatus”[tiab] OR “ovatus”[tiab]). Following the removal of duplicate studies, titles and abstracts were assessed by two independent researchers for relevance and eligibility for full-text review and study inclusion. Other studies were identified in bibliographies during full-text reviews of the studies retrieved. Additional studies and sources of tick surveillance data provided by academic experts in Japan were reviewed for inclusion. Academic experts in Japan shared their data and relayed the information that not much tick surveillance data are available in Japan. It was only when SFTS was first identified in Japan in 2013 (Liu et al., 2014) that more tick surveillance data were collected.

### 2.2 Inclusion criteria and data extraction

Studies with tick surveillance data associated with the following species were extracted by two independent researchers and included for modeling due to their significance for public health or relevant biology, phenology and feeding behaviors: *Ixodes ovatus*, *Ixodes persulcatus*, *Amblyomma testudinarium*, *Dermacentor bellulus*, *Dermacentor taiwanensis*, *Haemaphysalis megaspinosa*, *Haemaphysalis formosensis*, *Haemaphysalis flava*, *Haemaphysalis japonica*, *Haemaphysalis hystricis*, *Haemaphysalis kitaokai*, and *Haemaphysalis douglasi.* Data for tick nymphs or adults were included if they were collected by active surveillance of tick dragging and flagging or field trapping of relevant vertebrate hosts (e.g. rodent reservoirs of important human pathogens or large mammals critical for tick reproduction), or passive surveillance *via* mammalian roadkill or trapping. Studies were excluded if: (i) the surveillance activities occurred prior to 1990; (ii) no specific locality smaller than prefecture was provided (e.g. village, town, city, or municipality) or could be identified; (iii) surveillance activities could not validate specific location during collection (e.g. citizen science surveillance or migratory bird species of adventitious ticks); or (iv) incomplete or erroneous data could not be resolved.

The total number of ticks (dependent variable) collected by the original studies and data sources was extracted into a new data file in Microsoft Excel according to the following variables: tick species, tick life stage, collection year(s), collection method(s), GPS coordinates of collection site (if available), city and/or municipality, and prefecture. The entire data file used for the study is provided (Supplementary Table S1). For studies that did not provide the latitude/longitude or any details associated with the location of the tick surveillance sites, GPS coordinates were manually extracted by comparing the mapped locations in a figure (if provided) or the centroid of the smallest administrative locality (e.g. city, village, district, etc.) reported in the original source study *via* Google Maps. Despite varying spatial precision across geocoordinates of reported tick collection sites extracted in the datafile, this approach outweighed potential data heterogeneity by incorporating additional presence/absence data and broader geographical observations, ensuring sufficient coverage and sample power for model training.

Data for 26 abiotic environmental factors with biological relevance on the life cycles of *I*. *ovatus* or *I. persulcatus* and enzootic circulation of tick-borne pathogens were collated from publicly available databases and used as explanatory predictor variables for model training (Doi et al., 2018, 2021a; Hoshi et al., 2025; Iijima et al., 2025; Ito et al., 2024b; Shimizu et al., 2024). Weather variables were collected at daily intervals between 1990 and 2022 at a resolution of 1000 m from the Japanese Weather Association (Japan Weather Association, 2024). Data for “elevation” were obtained at a resolution of 1000 m from the National Spatial Planning and Regional Policy Bureau National Land Numerical Data (Japan Meteorological Agency, 2012). Fourteen landcover and habitat variables were collected at a spatial resolution of 500 m from the Ministry of the Environment of Japan, Seventh National Survey on the Natural Environment (2005–2009) (Ministry of the Environment of Japan, 2023). All abiotic variables extracted for model training were classified according to the predefined categories in their respective datasets by the original sources; no additional metrics were used for classification. The full list and details for all 26 abiotic predictor variables used for model development are provided in Table 1.

**Table 1:** Detailed list of the 26 abiotic variables and relevant sources included for the development of a Random Forests machine-learning model to predict habitat suitability of *Ixodes ovatus* and *Ixodes persulcatus* in Japan.

Category	Subcategory	Variable predictor	Interval	Year range	Scale (m)
Climate	Sunshine	Annual average global solar radiation	Mean daily global solar radiation/year	1990–2022	1000
		Annual total sunshine hours	Mean daily total sunshine hours/year	1990–2022	1000
	Precipitation	Annual precipitation	Mean daily precipitation/year	1990–2022	1000
		Deepest snowfall in December	Maximum snow level recorded/December	1990–2022	1000
		Deepest snowfall of the year	Maximum snow level recorded/year	1990–2022	1000
	Temperature	Annual maximum temperature	Mean daily maximum temperature/year	1990–2022	1000
		Annual minimum temperature	Mean daily minimum temperature/year	1990–2022	1000
		March average temperature	Mean daily temperature/March	1990–2022	1000
		January minimum temperature	Mean daily minimum temperature/January	1990–2022	1000
		Annual average temperature	Mean daily temperature/year	1990–2022	1000
		June maximum temperature	Mean daily maximum temperature/June	1990–2022	1000
Topography	Topography	Elevation	Static	2012	1000
Land cover	–	Built-up	Static	2005–2009	500
	–	ENF (evergreen needle-leaf forest)	Static	2005–2009	500
	–	DBF (deciduous broad-leaf forest)	Static	2005–2009	500
	–	EBF (evergreen broad-leaf forest)	Static	2005–2009	500
	–	Cropland	Static	2005–2009	500
	–	Paddy field	Static	2005–2009	500
	–	Grassland	Static	2005–2009	500
	–	DNF (deciduous needle-leaf forest)	Static	2005–2009	500
	–	Bare	Static	2005–2009	500
	–	Water bodies	Static	2005–2009	500
	–	Bamboo forest	Static	2005–2009	500
	–	Solar panel	Static	2005–2009	500
	–	No data	Static	2005–2009	500
	–	Unclassified	Static	2005–2009	500

### 2.3 Data processing and machine-learning algorithm development

Extracted geocoordinates associated with each tick species were prepared and mapped to their respective municipalities. Geocoordinates were then verified for accuracy according to the reported coordinates in the original studies. If these coordinates were still considered to be reported in error, the coordinates were mapped to the correct locality within the municipality and prefectures’ centroid land coordinates. All environmental raster files corresponding to the 26 explanatory variables were harmonized to a common target grid prior to feature extraction. We used a target resolution of 0.005° (*c.*500 m) and a common geographical extent covering Japan. For predictors originally available at coarser resolution (e.g. 1000 m climate/topography), values were mapped onto the target grid by assigning each target cell the value of the nearest source cell (nearest-neighbor). This approach preserves original values and avoids introducing interpolated extremes, while ensuring all predictors are aligned on the same grid for model training. All data harmonization and visualizations were conducted using Python 3.9 (released October 5, 2020; https://www.python.org/downloads/release/python-390/).

We employed a Random Forest ML model for its robustness in handling nonlinear relationships and interactions among explanatory variable features from multiple sources with heterogeneous data (Auret and Aldrich, 2012). Specifically, Random Forest ML models help mitigate risks of overfitting specific datasets or studies by aggregating predictions across multiple decision trees trained on bootstrapped subsets, reducing the influence of any single data source and improving generalizations across heterogeneous datasets compared to other predictive modeling approaches that cannot handle non-linear associations. To account for their unique relationships with the Japanese climate and landscapes, separate models were developed for *I. ovatus* and *I. persulcatus*. All ML model development, including feature scaling, Random Forest classification, hyperparameter tuning, and spatial output projections was performed with Python 3.9 using scikit-learn (v.1.2.2) (https://scikit-learn.org) (Pedrogosa et al., 2011).

To account for uneven distribution of extracted occurrence sites and varying abundances respective to each tick species, we incorporated pseudo-absence points during model development. Pseudo-absence is a common analytical approach in predictive modeling studies to more evenly spatially distribute and better balance the training dataset by minimizing overfitting of the reported presence sites and improving model performance (Walter et al., 2020; Noll et al., 2023; Kelly et al., 2025). Randomly distributed pseudo-absence points throughout Japan were introduced into the model such that the number of “pseudo-absence” points equaled the number of extracted *I. ovatus* or *I. persulcatus* “occurrence” points to minimize risks of overfitting if any tick surveillance data observations reported increased abundances for any species. For either *I. ovatus* or *I. persulcatus*, pseudo-absence points were then selected from grid cells where no individuals of that species had been recorded. This approach allows the model to sample locations lacking any reported occurrences of the tick species and ensured that the classifier learned to differentiate between presence and absence locations while limiting the impacts of surveillance bias as much as possible. Predicted spatial suitability of *I. ovatus* or *I. persulcatus* was then performed by the Random Forest ML algorithm at a spatial resolution of 500 m according to the 26 environmental variables and the data inputs for (i) the reported “occurrence” data for each tick species associated with a unique locality; and (ii) the observed number of ticks collected for each species at the occurrence site such that X observations of X ticks were found at the respective GPS site so that the observed tick abundances contributed proportionally during model training (e.g. magnitude of each tick species’ presence across occurrence sites); relative to their associated pseudo-absences introduced in the models. The training process involved hyperparameter tuning, where the modeled depth was adjusted to balance overall complexity and generalization ensuring optimal generalization with the unseen data (e.g. nonlinear associations between predicted habitat suitability and explanatory variables). We focused hyperparameter tuning on maximum tree depth, as it is the primary factor influencing overfitting and underfitting in random forest models (Schratz et al., 2019). Hyperparameters for the minimum threshold of sample occurrences/abundances to split a node, the minimum number of trees, and the maximum number of features considered at each split were left at default values, as increasing the number of trees beyond a certain threshold typically yields diminishing returns while substantially increasing computational cost (Probst et al., 2019). By constraining the depth of the trees, we aimed to reduce the likelihood of overfitting while maintaining strong predictive accuracy.

### 2.4 Feature selection, predictive outputs, and algorithm performance evaluation

Feature selection was critical to enhancing model interpretability and reducing multicollinearity bias among explanatory variables. The final subset of variables included for modeling was determined using Pearson’s correlation coefficient threshold of *R* = 0.95 and a statistical significance threshold of *P* = 0.01 to identify correlated pairwise explanatory variables. The primary method to calculate feature importance was mean decrease in impurity (MDI) based on the features’ average gain of splits in the Random Forest tree. Permutation importance was used as a secondary feature importance metric to evaluate the consistency of variable rankings as determined *via* MDI. The entire feature selection process to ensure that only the most relevant predictors contributed to the final classification. The land cover and habitat variables were assessed for feature importance *via* one-hot encoding by aggregating them *post-hoc* into separate “categorical” land cover variables and computing the weighted mean scores proportional to the frequency of each category. This approach was only used to report feature-importance contribution as an overall land cover summary, while retaining category-level predictors in the model. Any variables that had feature importance scores = 0 were dropped from the model. The trained model generated probability scores indicating the suitability of different geographical regions for tick presence. A dot-based heatmap for each model was generated using a subsample of 200,000 points distributed across Japan.

To reduce inflation of performance due to spatial autocorrelation, we used spatially-stratified cross-validation in which administrative districts were treated as groups (Pascoe et al., 2019). Districts were partitioned into ten (10%) folds, and in each iteration, all occurrence records within districts in the hold-out fold were withheld from model fitting and used only for evaluation. This group-based partitioning reduces spatial leakage between testing and training because nearby points within the same district are not split across folds. The primary evaluation metric was the area under the curve (AUC), which assessed the model’s ability to distinguish between presence and absence locations of the target species. AUC was calculated at the administrative district level, defining a district as “positive” for *Ixodes* spp. suitability if it had at least 30 individual ticks present (Estrada-Peña et al., 2018). To construct the AUC, we compared each district’s status to the maximum predicted risk value within that district, using 10-fold cross-validation to maintain spatial precision and avoid overfitting due to limited data. This administrative district-level approach helped mitigate spatial autocorrelation and reduced the impact of surveillance bias, where unsampled points may be incorrectly treated as true absences.

We aggregated predictions to the administrative district level for evaluation because this spatial scale provides a pragmatic, actionable spatial unit and reduces sensitivity to point-level sampling density and surveillance heterogeneity.

Lastly, to assess model stability and account for potential dilution of feature importance rankings among correlated variables based on MDI (due to their potential feature importance contributions distributed across multiple variables) (Pudjihartono et al., 2022), we re-ran the full modeling pipeline ten times using different random seeds while keeping the same feature set and cross-validation strategy. Across repeats, we summarized variability in AUC (mean and range and/or standard deviation) and assessed the consistency of predictor importance rankings (e.g. Spearman rank correlation of feature-importance vectors across runs). We also compared the resulting suitability surfaces qualitatively to confirm that high-suitability regions were robust to stochastic variation in bootstrap sampling and tree construction.

## 3 Results

### 3.1 Summary of data

**Fig. 1:**
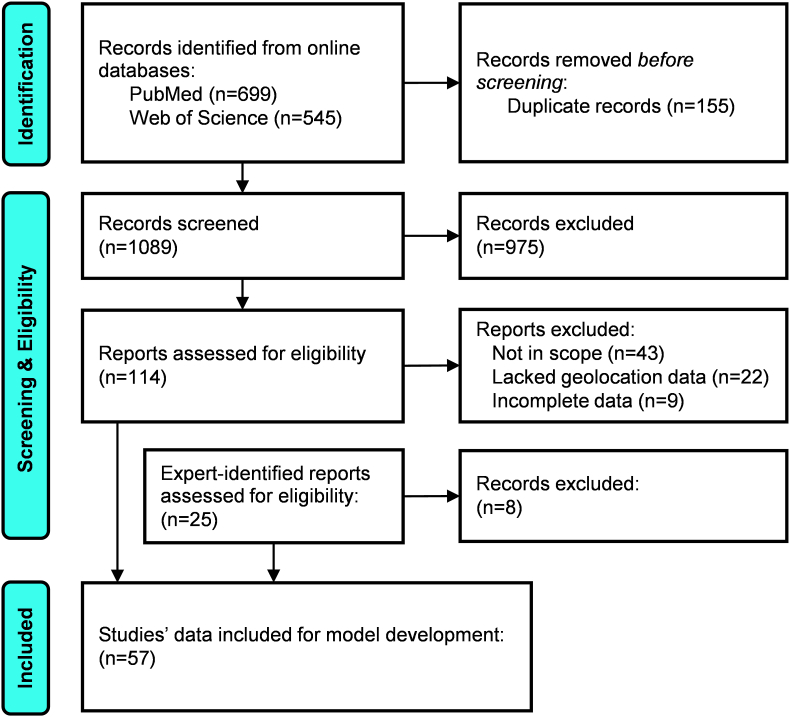
Flow diagram of the systematic literature review of retrieved studies and data sources for model development.

**Fig. 2:**
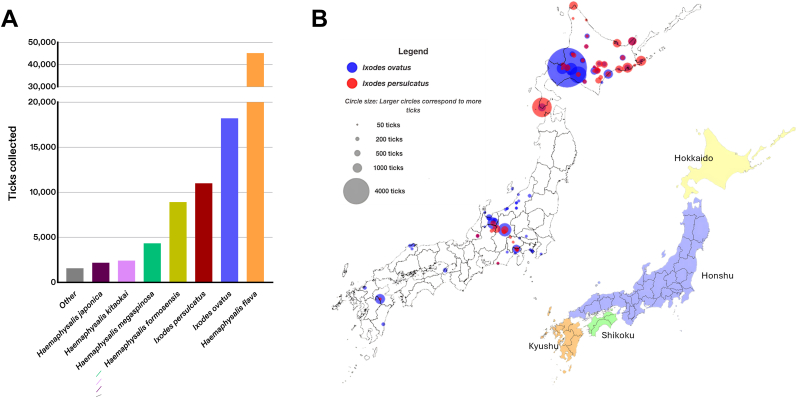
Summary of tick surveillance data extracted *via* systematic literature review for 11 tick vector species in Japan, 1990–2023. Data were aggregated into a database to predict habitat suitability of *Ixodes ovatus* and *Ixodes persulcatus* in Japan. **A** The total numbers of individual ticks extracted and considered for each species during the development of the Random Forest machine-learning (ML) algorithms are provided. **B** Map illustrating the occurrence and abundance data for *Ixodes ovatus* (*blue*) and *Ixodes persulcatus* (*red*) that were used as ML inputs are shown (data may not be exact). *Note*: the center of the circles corresponds to the approximate locality of the tick surveillance collection sites and the size of the circles illustrates the approximate abundance associated with the total number of ticks collected and extracted for analysis.

The results from the systematic literature review are provided (Fig. 1). After removal of duplicates, 1089 studies were retrieved from online databases *via* the search string; of these, 40 met the inclusion criteria. Data from another 17 studies were also extracted from an additional 25 studies or records provided by local experts for a total of 57 studies included in the study (Supplementary Table S2). Data for a total of 93,829 ticks across 11 species collected between 1991 and 2023 were extracted (Table 2, Fig. 2A). The most abundant species collected were *H. flava* (*n* = 45,198) followed by *I. ovatus* (*n* = 18,210) and *I. persulcatus* (*n* = 11,000) (Fig. 2A). Over half of the ticks included in the dataset were adults (*n* = 58,710) compared to nymphs (*n* = 32,891) (Table 2). Nearly all (96.1%) of the ticks were collected by active tick dragging or flagging (*n* = 55,865) or animal trapping (*n* = 34,350), while the remaining (*n* = 3614) were collected *via* passive animal collections of roadkill (*n* = 471) or animal trapping (*n* = 3143) (Table 3). Thirty-two (68%) of the 47 prefectures in Japan were represented, with the highest total tick counts in Kanagawa (*n* = 32,129) and Hokkaido (*n* = 24,878) (Table 4). Data were extracted from 29 prefectures for *I. ovatus* and 14 prefectures for *I. persulcatus*; both had the highest totals in Hokkaido, with 11,070 and 8839 ticks collected, respectively, for each species. The localities and abundances of *I. ovatus* and *I. persulcatus* across Japan extracted for the model are illustrated (Fig. 2B), and the occurrences of the nine non-*Ixodes* species that were extracted for database aggregation are provided as supplemental information (Supplementary Fig. S1).

**Table 2:** Total number of individual ticks per life stage and species extracted for analysis.

Species	Nymphs	Adults	Not reported	Total
*Ixodes ovatus*	60	17,211	939	18,210
*Ixodes persulcatus*	1444	8680	876	11,000
*Haemaphysalis flava*	19,042	26,156	–	45,198
*Haemaphysalis formosensis*	8106	809	–	8915
*Haemaphysalis megaspinosa*	2949	1289	100	4338
*Haemaphysalis kitaokai*	–	2418	–	2418
*Haemaphysalis japonica*	435	1642	103	2180
*Haemaphysalis hystricis*	312	331	–	643
*Haemaphysalis douglasi*	1	31	210	242
*Amblyomma testudinarium*	540	98	–	638
*Dermacentor taiwanensis*	2	45	–	47
Total	32,891	58,710	2228	93,829

**Table 3:** Total number of individual ticks collected by active and passive surveillance methods for eleven vector species.

Tick species	Active			Passive			Grand total
	Dragging/flagging	Animal trapping	Total	Animal trapping	Roadkill	Total	
*Ixodes ovatus*	15,494	2273	17,767	443		443	18,210
*Ixodes persulcatus*	10,289	171	10,460	540		540	11,000
*Haemaphysalis flava*	13,189	31,584	44,773	19	406	425	45,198
*Haemaphysalis formosensis*	8898	13	8911	4		4	8915
*Haemaphysalis megaspinosa*	3779	248	4027	254	57	311	4338
*Haemaphysalis kitaokai*	2418		2418				2418
*Haemaphysalis japonica*	575	40	615	28		28	643
*Haemaphysalis hystricis*	370	17	387	1793		1793	2180
*Haemaphysalis douglasi*	242		242				242
*Amblyomma testudinarium*	564	4	568	62	8	70	638
*Dermacentor taiwanensis*	47		47				47
Total	55,865	34,350	90,215	3143	471	3614	93,829

**Table 4:** Total number of individual ticks extracted for each species from Japan prefectures (1990–2023).

Prefecture	*I. ovatus*	*I. persulcatus*	*H. flava*	*H. formosensis*	*H. megaspinosa*	*H. kitaokai*	*H. japonica*	*H. hystricis*	*H. douglasi*	*A. testudinarium*	*D. taiwanensis*	Total
Kanagawa	147		31,601		305	56	2	1		16	1	32,129
Hokkaido	11,070	8839	448	36	2098	106	1986	26	242	27		24,878
Nagasaki	9	1	1037	5965	191	31		2		14		7250
Niigata	1344	25	2926		3		7				24	4329
Kagoshima	27		1106	1945	24	40		312		248	4	3706
Toyama	1767	343	1218		1	1	160					3490
Ehime			1902	398						77		2377
Nagano	1026	1142			2							2170
Miyazaki	129		123	505	56	1003		7		18	1	1842
Tokyo	2		478		1173	1						1654
Ibaraki	4		1525	1	6			56		27		1619
Shimane	215		368		272	629						1484
Shizuoka	786	340	90	3	4			9		11	1	1244
Oita	692	58	105			186						1041
Ishikawa	34	1	963					1		8	3	1010
Fukui	259	108	372		1	165	17			1	12	935
Nara	2	2	406		59	1				8		478
Okinawa				43				150		136		329
Hyogo	190		89									279
Yamaguchi	2		80		96	71						249
Yamanashi	99	117	3									219
Saitama	128		30			33						191
Kumamoto	23		43		6	78						150
Fukuoka	105		33									138
Yamagata	5	2	19	19	2		4	79		2		132
Kyoto			88			5				36	1	130
Fukushima	92	12	17		1	3	4					129
Shiga	2		58		38	9				7		114
Saga	2		53									55
Chiba	35									1		36
Tochigi	4		17							1		22
Gunma	10	10										20
Total	18,210	11,000	45,198	8915	4338	2418	2180	643	242	638	47	93,829

### 3.2 Model performance and variables associated with suitability

Four Random Forest models were developed for each *Ixodes* tick species with varying data inclusion criteria and algorithm training procedures to determine the best-performing model for habitat suitability predictions. Presence-only models that excluded abundance tick surveillance data had the lowest performances (AUCs < 0.76) while the models that included both presence and abundance data for each tick species had higher performance metrics (AUCs > 0.81) (Supplementary Table S3). Ultimately, the respective models with the highest performance metrics that were selected for habitat suitability predictions incorporated both presence and abundance tick surveillance data from all potential collection methods (tick dragging or flagging, animal trapping, and passively) yielding AUC scores for *I. ovatus* and *I. persulcatus* of 0.89 and 0.92, respectively (Supplementary Table S3).

**Fig. 3:**
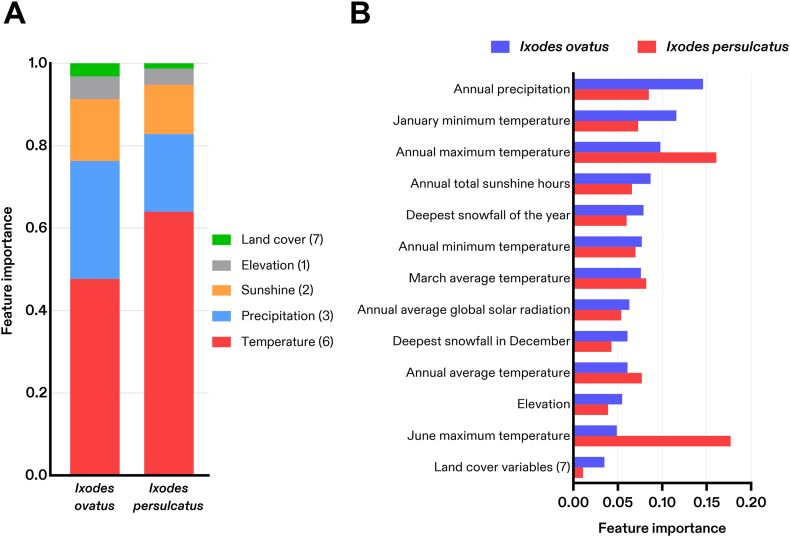
Individual (**A**) and categorical (**B**) abiotic feature importances of *Ixodes ovatus* and *Ixodes persulcatus* suitability. The 19 abiotic variables included in the final model were ranked according to their feature importances in the Random Forest machine-learning algorithm. Variables that were excluded from the model during feature selection or did not contribute to the final model are not shown.

The final models included 19 abiotic variables as explanatory predictors that were ranked according to their respective contributions (termed “feature importance”) to predict habitat suitability for each tick species (Supplementary Table S4). Seven explanatory predictors for the land cover variables representing areas of “deciduous needle-leaf forest”, “bare land”, “water bodies”, “bamboo forest”, “solar panels”, “no data”, and “unclassified” were dropped from the final models for both species (Supplementary Table S4). All climate-related predictors (*n* = 11) outranked the landcover-related predictors (*n* = 8) of suitability in the models (based on both feature importance metrics of MDI and permutation importance), though varying rankings and model contributions were observed for each species (Supplementary Table S4). Temperature-related variables (*n* = 7) accounted for six of the top seven-ranked predictors and contributed nearly two-thirds (0.640) of the predictive power in the suitability model for *I. persulcatus* but were responsible for less than half (0.477) of the model contributions for *I. ovatus* (Fig. 3A). Conversely, the precipitation variables (*n* = 3) and sunshine variables (*n* = 2) contributed more to the suitability model for *I. ovatus* (0.286 and 0.150) compared to *I. persulcatus* (0.188 and 0.120), respectively (Fig. 3A). Individually, the top-ranked predictor of suitability for *I. persulcatus* was “June maximum temperature” (0.177) which was the lowest-ranked (12th) explanatory predictor among climate variables for *I. ovatus* (0.049) (Fig. 3B). “Annual precipitation” (0.146) was the best predictor for *I. ovatus* followed by “January minimum temperature” (0.116), and “annual maximum temperature” (0.099). “Elevation” provided limited predictive power (< 0.055) as well as the collective landcover variables (*n* = 7) (< 0.035) in either model (Fig. 3A and B). Detailed results are for all abiotic variables’ feature importances to predict habitat suitability of *I. ovatus* and *I. persulcatus via* both metrics are provided in the supplemental information (Supplementary Table S4).

### 3.3 Predicted habitat suitability of *I. ovatus* and *I. persulcatus* in Japan

**Fig. 4:**
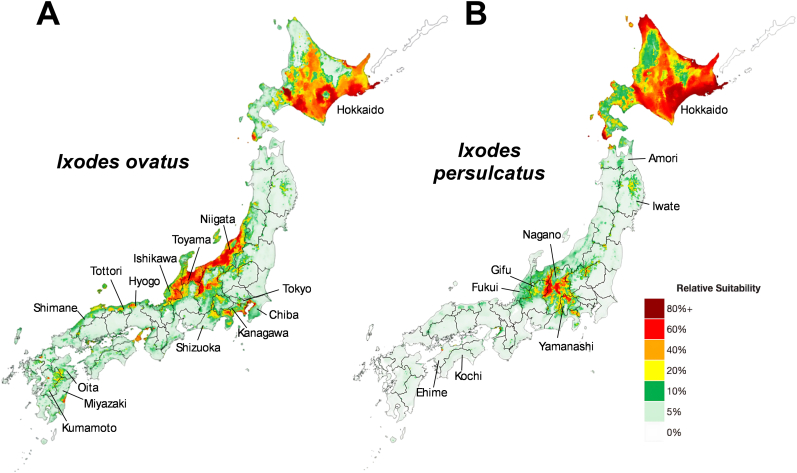
Predicted habitat suitability of *Ixodes ovatus* (**A**) and *Ixodes persulcatus* (**B**) in Japan. Outputs were developed using a Random Forest machine-learning algorithm according to 19 abiotic variables.

The model for *I. ovatus* predicted highly suitable (> 60%) habitat areas in southern and eastern regions of Hokkaido, in central Honshu, mainly in the northern parts of Niigata and Toyama and the mountainous areas in Ishikawa and Nagano surrounding northern Gifu (Fig. 4A). Sporadic suitability for *I. ovatus* was also predicted across Yamanashi and Shizuoka prefectures outside of Tokyo Bay in Kanagawa and Chiba (Fig. 4A). Moderate suitability (20–60%) for *I. ovatus* was predicted along the northern coast of southern Honshu in Hyogo, Tottori, and Shimane, and in central Kyushu between Oita, Kumamoto, and Miyazaki (Fig. 4A). For *I. persulcatus,* the model predicted high suitability limited within most of Hokkaido, with moderate suitability predicted through the southern peninsula south of Sapporo (Fig. 4B). Focal areas in the mountains of central Nagano and adjoining borders between Gifu and Toyama, and northern Yamanashi also had high predicted suitability of *I. persulcatus,* with spatially diffuse moderate predicted suitability from these regions (Fig. 4B).

## 4 Discussion

### 4.1 Summary of results

We leveraged an ML algorithm to predict suitable habitat areas for the occurrence of *I*. *ovatus* and *I*. *persulcatus* and identified the abiotic factors that predict their suitable habitat areas. Our analysis predicted highly suitable habitat areas outside of Hokkaido for both *I. ovatus* and *I. persulcatus*, with a broader distribution of *I. ovatus* further south in warmer and higher precipitation prefectures along the east coast of Honshu, while *I. persulcatus* prefers colder and drier environments at higher altitudes in central Honshu. *Ixodes ovatus* is also more likely to be found in hyper focal areas in subtropical areas of Kyushu, which is supported by the first reported Lyme disease and TBE cases in Oita prefecture (Ohira et al., 2023; NIID, 2025; Ohira et al., 2025).

Our results indicate that both *I. ovatus* and *I. persulcatus* may be more widespread throughout Japan, expanding the potential risk of Lyme disease and TBE to other prefectures than Hokkaido. Globally, *Ixodes* spp. are primarily forest-dwelling ticks that are highly susceptible to desiccation and mortality in hot, arid climates, which restricts their survival in southern latitudes to higher altitudes with increased cloud cover and lower temperatures (Kahl and Gray, 2023). Our models reinforce this biology with > 60% suitability predicted for both species in Hokkaido prefecture (the coldest region in Japan) and central Honshu in the high-altitudinal mountainous regions in Nagano, with increased cloud cover and cooler temperatures, with distinct focal suitability for *I. ovatus* throughout Toyama and Niigata, where annual precipitation is highest in mainland Japan. Despite endemic *I. persulcatus* populations observed in Aomori and Iwate prefectures, our models did not predict high suitability throughout northern Honshu (Miyamoto et al., 2000; Ishiguro et al., 2005). However, our model identified suitable habitat areas for *I. ovatus* in the tropical regions of Kyushu, indicating potential ecological conditions permissive for local autochthony among increased reported cases of Lyme disease in recent years (Ohira et al., 2023, 2025; Hoshi et al., 2025). Other areas with higher than anticipated predicted suitability for *I. ovatus* were the coastlines of Kanagawa, Tokyo, and Chiba prefectures, in the hotter climates east of the Tokyo metropolis, which is similar to the predicted suitability from another study *via* a Maximum Entropy (MaxEnt) model developed using variables for climate, land cover, and host species (Doi et al., 2021a). These three prefectures have previously reported human cases of Lyme disease, suggesting a local presence and persistence of humans exposed to *B. burgdorferi*-infected *Ixodes* vectors (NIID, 2025). The broadly predicted suitability *I. ovatus* in Japan demonstrates a wider ecological niche and potential competitive advantage against *I. persulcatus.* As shown in recent studies and possibly in part due to ongoing climate change, an increasing proportion of Lyme disease patients in Hokkaido have been bitten by *I. ovatus* compared to *I. persulcatus* over a nearly two-decade period (Hashimoto et al., 2002; Sasaki et al., 2021). Collectively, these results demonstrate the importance of understanding the geographical distribution and biological differences between *I. ovatus* and *I. persulcatus* to better inform public health surveillance for tick-borne diseases in Japan.

### 4.2 Expansion of tick vectors and tick-borne diseases in Japan

The potential for expanding distributions of *I. ovatus* and *I. persulcatus* across Japan is also recognized by the spread of other tick-borne diseases like Japanese spotted fevers and recent emergence of multiple novel tick-borne pathogens (Matsumoto et al., 2018; Sasaki et al., 2021; Ohira et al., 2023; Kumar et al., 2024; Ogawa et al., 2024; Ikeda et al., 2025). Among the most notable is SFTS virus (SFTSV), which led to an outbreak of 303 SFTS cases between 2013 and 2017 with a case-mortality rate of nearly 30% (Kobayashi et al., 2020). Data for several vector tick species with SFTSV detection were extracted in this study, such as *A. testudinarium*, *H. flava*, and *H. megaspinosa*. However, no data were collected on the species considered to be the primary vector in Japan, *H. longicornis*, due to its preferential feeding on domesticated animals, which is not consistent with the feeding habits of the other potential vectors and *I. ovatus* and *I. persulcatus* (Hayasaka et al., 2015; Matsumoto et al., 2018). Importantly, the emergence of potentially lethal tick-borne diseases like SFTS and Japanese spotted fevers has made the Japanese public aware of tick-borne diseases, underscoring the importance of ongoing surveillance of the geographical distribution and abundance of tick species over time to track vector distributions and pathogen circulation over time and ensure timely public notification of emerging threats.

The potential for continued expansion and increasing risk of tick-borne diseases may also be exacerbated by other environmental factors associated with important tick vectors, such as increasing temperatures and changing land-use patterns (Iijima et al., 2025). Rising temperatures caused by global climate change can extend active host-seeking periods by important tick vectors like the primary vector of Lyme disease in North America (*Ixodes scapularis*), which increases survival or accelerates their life cycle development (Ogden et al., 2018). Fragmented forests and specific habitat ecotones can also strongly (yet indirectly) increase tick-borne disease enzootic hazards by supporting optimal habitats for animal fauna with high reservoir competency (e.g. small rodents) that amplify tick-borne pathogen prevalence in local tick vectors (Moustafa et al., 2016; Takano et al., 2011). As climate change progresses in Japan, it is likely to exacerbate these trends, increasing the geographical range and incidence of tick-borne diseases. The continued long-term and regional-specific surveillance of *I. ovatus* and *I. persulcatus* is needed in Japan to understand how changing ecologies impact the geographical distribution and prevalence of tick-borne pathogens to support adaptive public health strategies that mitigate tick-borne disease risks.

### 4.3 Leveraging predictive algorithms to inform tick-borne disease risk

The increasing prevalence of tick-borne diseases highlights the importance of continuous surveillance and public education to reduce human encounters with infected tick populations and disease transmission. Many sources of surveillance data are passively collected for ticks (citizen science campaigns) and humans (local-, state-reported cases by public health departments) are lagging indicators of risk and disease burden, unable to provide information in ‘real-time’. Predictive models can sometimes more efficiently and reliably capture spatiotemporal trends of important tick vectors and associated tick-borne pathogens to prioritize surveillance efforts to identify potential risk areas (Bisanzio et al., 2020; Boligarla et al., 2023; Kiryluk et al., 2024). The majority of studies that have demonstrated the utility of predictive modeling to identify the ecological features associated with tick-borne diseases in Japan have focused on tick-borne pathogens with the potential to cause severe disease in humans, like SFTSV or Yezo virus (Ito et al., 2024a; Ogawa et al., 2024; Iijima et al., 2025) while relatively few have investigated environmental associations with *Ixodes* vector species and Lyme disease or TBE. Some predictive models for tick suitability may prefer other approaches like multiple mixed regression or MaxEnt, which are known for their simplicity, effectiveness, and interpretability compared to ML models (Warren and Seifert, 2011; Barbet-Massin et al., 2012; Estrada-Peña and de la Fuente, 2024; Kelly et al., 2025). We opted to use a Random Forest ML approach for its capacity to better handle potential biases introduced into the model *via* incorporation of data from multiple sources across regions with uneven sampling efforts in addition to its increased capacity to measure non-linear relationships with high-dimensional data across explanatory predictors and reduce the risk of overfitting. As an ensemble method, Random Forest approaches reduce overfitting and balance potential “noise” across heterogeneous inputs by averaging predictions across many trees. While we did not apply explicit weighting schemes, the strong AUC scores suggest that the model generalized well despite data source heterogeneity over space and time. We further demonstrated the utility of the Random Forest model by our approach to refitting the model repeatability, suggesting that the models were robust and not meaningfully affected by the presence of correlated features, providing confidence in the reliability of both our variable selection process and model outcomes. Although our ML model provided rankings of the explanatory predictors that best explain habitat suitability based on their individual feature importances, the Random Forest algorithm does not natively support traditional coefficients for positive or negative association like a linear model. Despite its strengths compared to MaxEnt, Random Forest models can be computationally intensive, although studies have been effectively utilized both to predict the spatial distribution of tick vectors, highlighting the practical applications and limitations of each method (Doi et al., 2021a; Kelly et al., 2025).

### 4.4 Limitations

Nevertheless, our analysis has some limitations. First, we recognize the increased data heterogeneity and variance in our dataset for model development, which is expected during meta-analyses (Kriston, 2013). The extracted tick surveillance data for model training were collected by multiple studies using varying methods over a broad temporal period. Although studies were identified using broad search terms in two online databases, bibliography review of included studies, and provided by project collaborators, there is a chance that relevant data were not identified. Available tick surveillance data are lacking in areas of Japan with poor accessibility, such as northern Honshu, known to be suitable habitat area of *I. persulcatus* (NIID, 2025), leading to model “blinding” to predict suitability in similar habitat areas across the country. Further, some of the original studies did not meet the inclusion criteria because we could not verify the tick surveillance collection sites, despite available geocoordinates reported by the original studies in some cases, which would have increased model precision. Model bias may also be influenced by species misidentification of *I. ovatus* or *I. persulcatus* with other species like *Ixodes nipponensis* that have different ecological preferences and host-feeding habits (Kitaoka and Saito, 1967). Furthermore, some of the extracted data for *I. ovatus* or *I. persulcatus* occurred at collection sites that are not considered suitable habitat areas for either species, such as the hotter climates of the greater Tokyo metropolis, which would benefit from confirmation of locally established, reproducing tick populations by additional tick surveillance studies.

It is also possible our ML algorithm could have benefited from the inclusion of epidemiological surveillance data associated with other tick species or human case reports of Lyme disease and TBE. The availability of human epidemiological surveillance data is limited due to diagnostic test kits with suboptimal capacities to diagnose TBE or Lyme disease in clinical settings, which makes it more challenging to conduct surveillance across the country and reduces understanding of the geographical distribution of both diseases (Ohira et al., 2023, 2025). We note, however, that our model showed high performance characteristics when excluding input data associated with other tick species that exhibit less similar phenology traits, host-seeking behaviors, and feeding preferences than *I. ovatus* and *I. persulcatus* (Kitaoka and Saito, 1967; Yamane et al., 2006). We acknowledge that habitat suitability for animal species can be predicted by presence-only data without incorporating the proportional magnitude for the observed occurrences, which could reduce data bias, but we did not have sufficient data for *I. ovatus* and *I. persulcatus* to effectively power our model for accurate predictions. Additionally, our models were developed using data with inconsistent spatial scales and varying precisions of extracted geocoordinates where ticks were collected based on the available information provided in the original studies. Importantly, we were able to successfully harmonize the data across variables and studies as data inputs with varying spatial scales (both dependent and independent variables) are commonly incorporated into modeling studies for tick-borne diseases when predictions are made across large geographies like Europe (Kelly et al., 2025) and the USA (Burtis et al., 2022). Despite the potential of increased uncertainty in these modeled outputs, we felt the decision to incorporate heterogeneous data from multiple studies with varying collection methods or spatial and temporal scales was a practical compromise that outweighed potential pitfalls in data bias by ensuring sufficient geographical coverage and sample power when training the ML algorithms.

Lastly, although abiotic variables are important for tick ecosystems and are suitable predictive variables, we did not incorporate biotic variables such as the distribution of tick vertebrate hosts and reservoirs because of the broad spatial scales (25 km^2^ gridded cells) of mammalian species provided by the Ministry of the Environment in Japan, which we felt was too poor a resolution for inclusion in the model. However, we recognize that the inclusion of biotic variables may have strongly contributed to the models’ predictions as demonstrated by other predictive modeling studies of tick-borne diseases in Japan (Ito et al., 2024a, 2024b). Different important tick vector species have varying host preferences and feeding habits; thus, we suspect the inclusion of the expected distributions of certain host species would provide strong contributions during model development. Future iterations of the model, leveraging temporally dynamic expected distributions of important tick hosts and reservoir species concurrently with relevant abiotic variables are planned to further strengthen suitability predictions of both *I. ovatus* and *I. persulcatus* and other important tick vector species in Japan.

## 5 Conclusions

In summary, this study shows that suitable habitat for key *Ixodes* vectors is likely more widespread in Japan than previously recognized, suggesting ongoing geographical expansion. Our findings demonstrate the value of machine-learning approaches for improving understanding of the distribution and ecological drivers of *I. ovatus* and *I. persulcatus*. Continued interdisciplinary research is essential to address the growing challenges of tick-borne diseases, particularly under changing climatic conditions. Strengthening predictive modeling and public health responses can improve risk assessment, preparedness, and prevention, ultimately supporting better control of Lyme disease, TBE, and other tick-borne threats in Japan.

## Ethical approval

Not applicable.

## CRediT authorship contribution statement

**Patrick H. Kelly:** Conceptualization, Writing - original draft, Writing - review & editing, Formal analysis, Data visualization. **Harrison M. Marick:** Formal analysis, Data visualization, Writing - review & editing; **Julie Davis:** Data curation, Writing - review & editing; **Ai Takano:** Data curation, Conceptualization, Analytical design, Writing - review & editing. **Kentaro Yoshii:** Conceptualization, Writing - review & editing. **Hiroki Kawabata:** Data curation. Analytical design, Writing - review & editing. **Kentaro Itokawa:** Data curation, Writing - review & editing. **Kozue Sato:** Data curation, Writing - review & editing. **Alice C.C. Lau:** Data curation, Writing - review & editing. **Yongjin Qiu:** Data curation, Writing - review & editing. **Frederick J. Angulo**: Writing - review & editing. **Andreas Pilz:** Supervision, Funding acquisition, Writing - review & editing. **Agustín Estrada-Peña:** Analytical design, Writing - review & editing, Data visualization. **Shuhei Ito:** Conceptualization, Writing - review & editing. **Jennifer C. Mo**ï**si:** Funding acquisition, Supervision, Writing - review & editing. **Yoshikazu Nakayama:** Conceptualization, Data curation, Writing - original draft, Writing - review and editing.

## Funding

This study was sponsored by 10.13039/100004319Pfizer.

## Declaration of competing interests

Patrick H. Kelly, Andreas Pilz, Frederick J. Angulo, Shuhei Ito, Jennifer C. Moïsi and Yoshikazu Nakayama are employees of Pfizer, Inc. and may hold shares and/or stock options in the company. Harrison M. Marick, Agustín Estrada-Peña, and Julie Davis are paid consultants for Pfizer, Inc. in connection with the development of this manuscript. Pfizer, Inc. owns a tick-borne encephalitis vaccine (FSME-IMMUN©/Ticovac©). The other authors declare that they have no known competing financial interests or personal relationships that could have appeared to influence the work reported in this paper. Given their role as Co-Editor, Agustín Estrada-Peña had no involvement in the peer review of this article and has no access to information regarding its peer review. Full responsibility for the editorial process for this article was delegated to Professor Aneta Kostadinova (Editor-in-Chief).

## Data Availability

All data used for this study are described and provided in the article and its supplementary files.
