# Supplementary material for: Detection of suitable habitat areas in Japan of the Lyme disease and tick-borne encephalitis vectors Ixodes ovatus and Ixodes persulcatus based on abiotic factors
Source: Curr Res Parasitol Vector Borne Dis. 2026 May 14;9:100385. doi: 10.1016/j.crpvbd.2026.100385 (PMC13223795; doi:10.1016/j.crpvbd.2026.100385)
Supplement: Multimedia component 2 [file mmc2.pdf]

Supplementary Table S2. Detailed list of data sources and references included in the study.

#	REFID	Citation_source
1	Arai, 2021	Arai R, Sato M, Kato M, et al. Spotted fever group rickettsiae (SFGR) detection in ticks following reported human case of Japanese spotted fever in Niigata Prefecture, Japan. *Sci Rep*. 2021;11(1):2595. doi:10.1038/s41598-021-81587-9
2	Azama, 2021	Azama Y, Fukuchi Y, Kuba Y, Kyan H. Seasonal occurrence of ixodid ticks on the vegetation in the northern part of Okinawajima Island, Japan. *Med Entomol Zool*. 2021;72:27-30. doi: 10.7601/mez.72.27
3	Azama, 2022	Azama Y, Kuba Y, Kyan H, et al. Ixodid ticks recovered from various animals on Okinawajima Island, Japan, in the period 2016-2021. *Med Entomol Zool*. 2022;73(2):63-67. doi: 10.7601/mez.73.63
4	Doi, 2018	Doi K, Kato T, Hayama SI. Infestation of introduced raccoons (*Procyon lotor*) with indigenous ixodid ticks on the Miura Peninsula, Kanagawa Prefecture, Japan. *Int J Parasitol Parasites Wildl*. 2018;7(3):355-359. doi:10.1016/j.ijppaw.2018.09.002
5	Doi, 2020	Doi K, Nishida K, Kato T, Hayama SI. Effects of introduced sika deer (*Cervus nippon*) and population control activity on the distribution of Haemaphysalis ticks in an island environment. *Int J Parasitol Parasites Wildl*. 2020;11:302-307. doi:10.1016/j.ijppaw.2020.03.001
6	Doi, 2021	Doi K, Kono M, Kato T, Hayama SI. Ecological traps and boosters of ixodid ticks: The differing ecological roles of two sympatric introduced mammals. *Ticks Tick Borne Dis*. 2021;12(3):101687. doi:10.1016/j.ttbdis.2021.101687
7	Fujita, 1996	Fujita H, Takada N, Tsuboi Y. Survey of ixodid ticks (Acarina : Ixodidae) and tick-borne spotted fever group rickettsiae in Tokunoshima Island, Japan. *Med Entomol Zool*. 1996;47(1):15-21. doi: 10.7601/mez.47.15
8	Furuno, 2017	Furuno K, Lee K, Itoh Y, et al. Epidemiological study of relapsing fever borreliae detected in Haemaphysalis ticks and wild animals in the western part of Japan. *PLoS One*. 2017;12(3):e0174727. doi:10.1371/journal.pone.0174727
9	Hoshi, 2025	Hoshi T, Ishigaki E, Khongyot T, Kaneko S. Ecological overview of hard ticks (Ixodida: Ixodidae) in Nagasaki prefecture of western Japan during winter 2021-2022. *Sci Rep*. 2025;15(1):4114. doi:10.1038/s41598-025-87085-6
10	Inayoshi, 2004	Inayoshi M, Naitou H, Kawamori F, Masuzawa T, Ohashi N. Characterization of Ehrlichia species from Ixodes ovatus ticks at the foot of Mt. Fuji, Japan. *Microbiol Immunol*. 2004;48(10):737-745. doi:10.1111/j.1348-0421.2004.tb03599.x
11	Inokuma, 2002	Inokuma H, Fujimoto T, Hosoi E, et al. Tick infestation of sika deer (*Cervus nippon*) in the western part of Yamaguchi Prefecture, Japan. *J Vet Med Sci*. 2002;64(7):615-617. doi:10.1292/jvms.64.615
12	Ishiguro, 1992	Ishiguro F, Iida H, Hatano M, Yano Y, Takada N. Tick fauna and the prevalence of Lyme Borrelia in Fukui Prefecture. *J Acarol Soc Jpn*. 1992;1(1):27-35. doi: 10.2300/acari.1.27
13	Ishiguro, 2008	Ishiguro F, Takada N, Fujita H, Noji Y, Yano Y, Iwasaki H. Survey of the vectorial competence of ticks in an endemic area of spotted fever group rickettsioses in Fukui Prefecture, Japan. *Microbiol Immunol*. 2008;52(6):305-309. doi:10.1111/j.1348-0421.2008.00042.x
14	Ito, 2024a	Ito M, Minamikawa M, Kovba A, et al. Environmental and host factors underlying tick infestation in invasive raccoons (Procyon lotor) in Hokkaido, Japan. *Ticks Tick Borne Dis*. 2024;15(6):102389. doi:10.1016/j.ttbdis.2024.102389

#	REFID	Citation_source
15	Ito, 2024b	Ito M, Minamikawa M, Kovba A, et al. Environmental and host factors underlying tick-borne virus infection in wild animals: Investigation of the emerging Yezo virus in Hokkaido, Japan. *Ticks Tick Borne Dis*. 2024;15(6):102419. doi:10.1016/j.ttbdis.2024.102419
16	Jamsransuren, 2019	Jamsransuren J, Yoshii K, Kariwa H, Asakawa M, Okuda K, Fujii K, et al. Epidemiological survey of tick-borne encephalitis virus infection in wild animals on Hokkaido and Honshu islands, Japan. *Jpn J Vet Res*. 67(2):163-172. doi:10.14943/jjvr.67.2.163
17	Kishimoto, 2024	Kishimoto M, Itakura Y, Tabata K, et al. A wide distribution of Beiji nairoviruses and related viruses in Ixodes ticks in Japan. *Ticks Tick Borne Dis*. 2024;15(6):102380. doi:10.1016/j.ttbdis.2024.102380
18	Kiyasu, 2024	Kiyasu Y, Osawa S, Tsutsumi N, Terada N, Nagata N. Distribution of ticks and their possession of spotted fever group Rickettsia in Ibaraki prefecture. *J Infect Chemother*. 2024;30(7):590-596. doi:10.1016/j.jiac.2023.12.013
19	Kobayashi, 2021	Kobayashi D, Kuwata R, Kimura T, et al. Detection of Jingmenviruses in Japan with Evidence of Vertical Transmission in Ticks. *Viruses*. 2021;13(12):2547. doi:10.3390/v13122547
20	Kobayashi, 2022	Kobayashi D, Kuwata R, Kimura T, et al. Detection of Japanese Encephalitis Virus RNA in Host-Questing Ticks in Japan, 2019-2020. *Am J Trop Med Hyg*. Published online April 11, 2022. doi:10.4269/ajtmh.21-0700
21	Komine, 2023	Komine H, Okabe K. Summer collection of multiple southern species of ticks in a remote northern island in Japan and literature review of the distribution and avian hosts of ticks. *Exp Appl Acarol*. 2023;90(3-4):357-374. doi:10.1007/s10493-023-00819-x
22	Kuba, 2022	Kuba Y, Azama Y, Kyan H, et al. Severe Fever with Thrombocytopenia Syndrome Virus RNA in Ticks from Wild Mongooses in Okinawa Prefecture, Japan. *Jpn J Infect Dis*. 2022;75(6):612-615. doi:10.7883/yoken.JJID.2021.808
23	Kurita, 1995	Kurita T, Kawabata H, Yamada K, et al. [Prevalence of Lyme Borrelia on tick collected in Shizuoka prefecture]. *Kansenshogaku Zasshi*. 1995;69(3):324-326. doi:10.11150/kansenshogakuzasshi1970.69.324
24	Matsumoto, 2018	Matsumoto N, Masuoka H, Hirayama K, Yamada A, Hotta K. Detection and phylogenetic analysis of phlebovirus, including severe fever with thrombocytopenia syndrome virus, in ticks collected from Tokyo, Japan. *J Vet Med Sci*. 2018;80(4):638-641. doi:10.1292/jvms.17-0604
25	Matsumura, 2024a	Matsumura R, Kobayashi D, Itoyama K, Isawa H. Detection of novel coltivirus-related sequences in Haemaphysalis megaspinosa ticks collected from Kanagawa Prefecture, Japan. *J Vet Med Sci*. 2024;86(8):866-871. doi:10.1292/jvms.24-0124
26	Matsumura, 2024b	Matsumura R, Kobayashi D, Itoyama K, Isawa H. First Detection of the Jingmen Tick Virus in Amblyomma testudinarium Ticks from the Kanto Region, Japan. *Jpn J Infect Dis*. 2024;77(3):174-177. doi:10.7883/yoken.JJID.2023.347
27	Matsuyama, 2023	Matsuyama H, Doi K, Agetsuma N, Suzuki M. Quantifying the direct and indirect effects of sika deer (Cervus nippon) on the prevalence of infection with Rickettsia in questing Haemaphysalis megaspinosa: A field experimental study. *Ticks Tick Borne Dis*. 2023;14(5):102201. doi:10.1016/j.ttbdis.2023.102201
28	Mekata, 2023	Mekata H, Kobayashi I, Okabayashi T. Detection and phylogenetic analysis of Dabieshan tick virus and Okutama tick virus in ticks collected from Cape Toi, Japan. *Ticks Tick Borne Dis*. 2023;14(6):102237. doi:10.1016/j.ttbdis.2023.102237

#	REFID	Citation_source
29	Miyamoto, 1992	Miyamoto K, Nakao M, Fujimoto K, Yamaguti N, Hori E. Detection of *Borrelia burgdorferi* in ixodid ticks collected from the Chichibu mountainous region of Central Honshu, Japan. *Med Entomol Zool*. 1992;43(3):255-258. doi:10.7601/mez.43.255
30	Miyamoto, 1997	Miyamoto K, Sato Y, Okada K, Fukunaga M, Sato F. Competence of a migratory bird, red-bellied thrush (*Turdus chrysolaus*), as an avian reservoir for the Lyme disease spirochetes in Japan. *Acta Trop*. 1997;65(1):43-51. doi:10.1016/s0001-706x(97)00651-7
31	Nakao, 1996	Nakao M, Uchikawa K, Dewa H. Distribution of Borrelia species associated with Lyme disease in the subalpine forests of Nagano prefecture, Japan. *Microbiol Immunol*. 1996;40(4):307-311. doi:10.1111/j.1348-0421.1996.tb03351.x
32	Nakao, 1997	Nakao M, Takada N. Survey of tick fauna in the Kyushu mainland, Japan. *Med Entomol Zool*. 1997;48(1):39-44.
33	Noda, 2006	Noda S, Yamamoto S. Detection of Japanese spotted fever rickettsiae DNA from ixodid ticks in Osumi Peninsula of Kagoshima Prefecture, Japan. *Med Entomol Zool*. 2006;57(4):273-277. doi:10.7601/mez.57.273
34	Ohashi, 2005	Ohashi N, Inayoshi M, Kitamura K, et al. Anaplasma phagocytophilum-infected ticks, Japan. *Emerg Infect Dis*. 2005;11(11):1780-1783. doi:10.3201/eid1111.050407
35	Okado, 2021	Okado K, Adjou Moumouni PF, Lee SH, et al. Molecular detection of *Borrelia burgdorferi* (*sensu lato*) and *Rickettsia* spp. in hard ticks distributed in Tokachi District, eastern Hokkaido, Japan. *Curr Res Parasitol Vector Borne Dis*. 2021;1:100059. doi:10.1016/j.crpvbd.2021.100059
36	Regilme, 2021	Regilme MAF, Sato M, Tamura T, et al. Comparative population genetic structure of two ixodid tick species (Acari:Ixodidae) (Ixodes ovatus and Haemaphysalis flava) in Niigata prefecture, Japan. *Infect Genet Evol*. 2021;94:104999. doi:10.1016/j.meegid.2021.104999
37	Sakai, 2014	Sakai A, Torii H. Tick fauna collected from the carcasses of sika deer in Nara park, central Japan. Bulletin of the Center for Natural Environment Education, Nara University of Education. 2014;15:27-34.
38	Sato, 2021	Sato M, Ikeda S, Arai R, et al. Diversity and distribution of ticks in Niigata prefecture, Japan (2016-2018): Changes since 1950. *Ticks Tick Borne Dis*. 2021;12(3):101683. doi:10.1016/j.ttbdis.2021.101683
39	Kawabata, 2013	Tick surveillance dataset conducted on Hokkaido Prefecture (2013), provided by Dr. Hiroki Kawabata, National Institute of Infectious Diseases, Japan.
40	Shibata, 2018	Shibata S, Sivakumar T, Igarashi I, et al. Epidemiological survey of a cervine Theileria in wild deer, questing ticks, and cattle in Hokkaido, Japan. *Ticks Tick Borne Dis*. 2018;9(5):1235-1240. doi:10.1016/j.ttbdis.2018.05.006
41	Shimizu, 2024	Shimizu K, Shimozuru M, Yamanaka M, Ito G, Nakao R, Tsubota T. Seasonal infestation patterns of ticks on Hokkaido sika deer (*Cervus nippon yesoensis*). *Parasitology*. doi:10.1017/S0031182024001227
42	Sivakumar, 2014	Sivakumar T, Tattiyapong M, Okubo K, et al. PCR detection of Babesia ovata from questing ticks in Japan. *Ticks Tick Borne Dis*. 2014;5(3):305-310. doi:10.1016/j.ttbdis.2013.12.006
43	Someya, 2015	Someya A, Ito R, Maeda A, Ikenaga M. Detection of rickettsial DNA in ticks and wild boars in Kyoto City, Japan. *J Vet Med Sci*. 2015;77(1):37-43. doi:10.1292/jvms.14-0451
44	Tabara, 2011	Tabara K, Kawabata H, Arai S, et al. High incidence of rickettsiosis correlated to prevalence of Rickettsia japonica among Haemaphysalis longicornis tick. *J Vet Med Sci*. 2011;73(4):507-510. doi:10.1292/jvms.10-0369

#	REFID	Citation_source
45	Takahashi, 2020	Takahashi Y, Kobayashi S, Ishizuka M, et al. Characterization of tick-borne encephalitis virus isolated from a tick in central Hokkaido in 2017. *J Gen Virol*. 2020;101(5):497-509. doi:10.1099/jgv.0.001400
46	Takano, 2022	Tick surveillance dataset conducted across multiple Japanese Prefectures (2022), provided by Dr. Ai Takano, Yamaguchi University, Japan.
47	Takano, 2014	Takano A, Toyomane K, Konnai S, et al. Tick surveillance for relapsing fever spirochete *Borrelia miyamotoi* in Hokkaido, Japan. *PLoS One*. 2014;9(8):e104532. doi:10.1371/journal.pone.0104532
48	Tsunoda, 2014	Tsunoda T. Detachment of hard ticks (Acari: Ixodidae) from hunted sika deer (*Cervus nippon*). *Exp Appl Acarol*. 2014;63(4):545-550. doi:10.1007/s10493-014-9795-x
49	Uchikawa, 1993	Uchikawa K. Seasonal fluctuations of Ixodes persulcatus and adult stage of Ixodes ovatus in the subalpine forests of Nagano Prefecture, Japan, related to observed phenological data (Acari, Ixodidae). *Med Entomol Zool*. 1993;44(3):203-211. doi:10.7601/mez.44.203
50	Yamauchi, 2009	Yamauchi T, Obara M, Watanabe M, Ando S, Ishikura M, Shinagawa Y, et al. Survey of tick fauna possessing the ability to act as vectors of rickettsiosis in Toyama Prefecture, Japan. *Med Entomol Zool*. 2009;60:23-31. doi:10.7601/mez.60.23
51	Yamauchi, 2020a	Yamauchi T, Watanabe M, Sawabe K. Survey of tick fauna in Ishikawa Prefecture, Japan (2013-2014). *JSMEZ*. 2020;71(2):101-104. doi: 10.7601/mez.71.101
52	Yamauchi, 2020b	Yamauchi T, Watanabe M, Sawabe K. Survey of tick fauna in Shiga Prefecture, Japan in 2014. *J Acarol Soc Jpn*. 2020;29(1):9-12. doi: 10.2300/acari.29.9
53	Yokoyama, 2012	Yokoyama N, Sivakumar T, Ota N, et al. Genetic diversity of Theileria orientalis in tick vectors detected in Hokkaido and Okinawa, Japan. *Infect Genet Evol*. 2012;12(8):1669-1675. doi:10.1016/j.meegid.2012.07.007
54	Yoshimoto, 2010	Yoshimoto K, Matsuyama Y, Matsuda H, et al. Detection of Anaplasma bovis and Anaplasma phagocytophilum DNA from Haemaphysalis megaspinosa in Hokkaido, Japan. *Vet Parasitol*. 2010;168(1-2):170-172. doi:10.1016/j.vetpar.2009.10.008
55	Zamoto-Niikura, 2012	Zamoto-Niikura A, Tsuji M, Qiang W, Nakao M, Hirata H, Ishihara C. Detection of two zoonotic Babesia microti lineages, the Hobetsu and U.S. lineages, in two sympatric tick species, ixodes ovatus and Ixodes persulcatus, respectively, in Japan. *Appl Environ Microbiol*. 2012;78(9):3424-3430. doi:10.1128/AEM.00142-12
56	Zamoto-Niikura, 2018	Zamoto-Niikura A, Tsuji M, Qiang W, et al. The Babesia divergens Asia Lineage Is Maintained through Enzootic Cycles between Ixodes persulcatus and Sika Deer in Hokkaido, Japan. *Appl Environ Microbiol*. 2018;84(7):e02491-17. doi:10.1128/AEM.02491-17
57	Zamoto-Niikura, 2023	Zamoto-Niikura A, Saigo A, Sato M, et al. The presence of Ixodes pavlovskyi and I. pavlovskyi-borne microorganisms in Rishiri Island: an ecological survey. *mSphere*. 2023;8(6):e0021323. doi:10.1128/msphere.00213-23

Supplementary Figure S1. General occurrences of the nine non-*Ixodes* tick species data extracted in the study. Maps illustrate the spatial occurrence data of the individual ticks for each species that were considered during model development. A) *Haemaphysalis flava*; B) *Haemaphysalis formosensis*; C) *Haemaphysalis megaspinosa*; D) *Haemaphysalis kitaokai*; E) *Haemaphysalis japonica*; F) *Haemaphysalis hystricis;* G) *Amblyomma testudinarium*; H) *Haemaphysalis douglasi*; and I) *Dermacentor taiwanensis*.

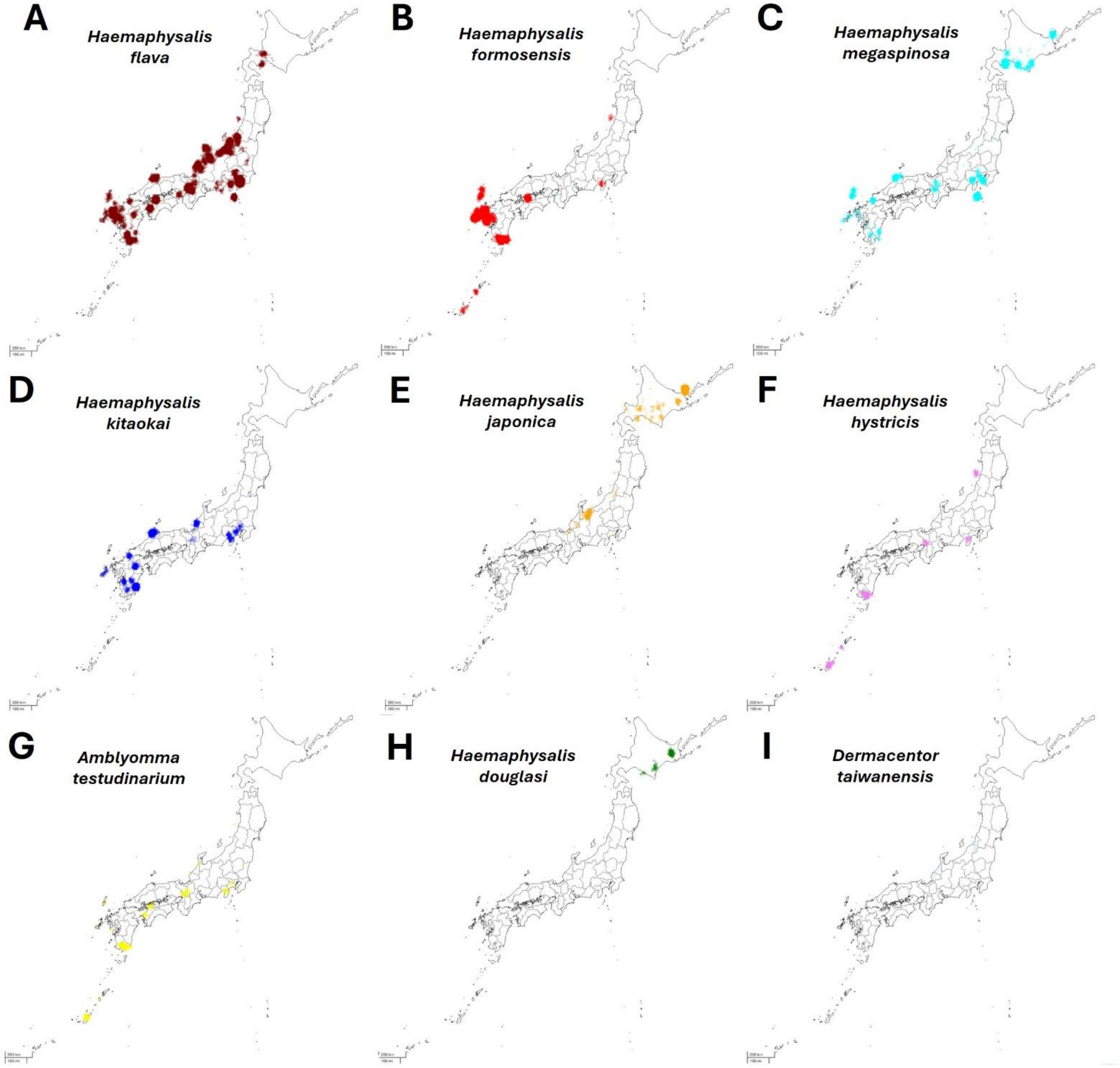

Supplementary Table S3. Performance evaluation of four Random Forest machine-learning models to predict habitat suitability of *Ixodes ovatus* and *Ixodes persulcatus* in Japan. Models were developed using varying inclusion criteria and output approaches and were assessed via Area Under the Curve (AUC) metric. *Denotes final version of the model used for predictive outputs of habitat suitability.

			Ixodes ovatus			***Ixodes persulcatus***		
**Model version**	**Data inputs**	**Study collection methods**	**No. observations**	**N ticks**	**AUC**	**No. observations**	**N ticks**	**AUC**
1	Tick occurrence	Tick dragging & flagging; Animal trapping; Passive surveillance	367	0	0.59	219	0	0.59
2	Tick occurrence & abundance	Tick dragging & flagging	357	15,495	0.84	214	10,289	0.81
3	Tick occurrence	Tick dragging & flagging; Animal trapping; Passive surveillance	367	0	0.74	219	0	0.76
4*	Tick occurrence & abundance	Tick dragging & flagging; Animal trapping; Passive surveillance	367	18,210	0.92	219	11,000	0.89

Supplementary Table S4. Feature importances and rankings of the abiotic variables in the final Random Forest machine-learning models to predict habitat suitability of *Ixodes ovatus* and *Ixodes persulcatus* in Japan according to Mean Decrease in Impurity (MDI) and Permutation Importance (PI) methods. Variables with "-" as their rank were either dropped from the model or did not contribute to the model outputs (e.g., feature importance equal to zero).

	Ixodes ovatus		*Ixodes persulcatus*		*Ixodes ovatus*		*Ixodes persulcatus*	
**Abiotic variable predictor**	**MDI**	**Rank**	**MDI**	**Rank**	**PI**	**Rank**	**PI**	**Rank**
Annual precipitation	0.146	1	0.085	3	0.082	1	0.033	1
January minimum temperature	0.116	2	0.073	6	0.022	3	0.011	8
Annual maximum temperature	0.098	3	0.161	2	0.009	10	0.017	4
Annual total sunshine hours	0.087	4	0.066	8	0.036	2	0.023	3
Deepest snowfall of the year	0.079	5	0.060	9	0.021	4	0.011	9
Annual minimum temperature	0.077	6	0.070	7	0.014	7	0.011	7
March average temperature	0.076	7	0.082	4	0.009	11	0.007	12
Annual average global solar radiation	0.063	8	0.054	10	0.017	5	0.015	5
Deepest snowfall in December	0.061	9	0.043	11	0.006	12	0.007	11
Annual average temperature	0.061	9	0.077	5	0.014	8	0.008	10
Elevation	0.055	11	0.039	12	0.015	6	0.012	6
June maximum temperature	0.049	12	0.177	1	0.014	9	0.028	2
Built-up	0.013	13	0.002	14	0.003	13	0.001	13
ENF (evergreen needle-leaf forest)	0.010	14	0.003	13	0.002	14	<0.001	14
DBF (deciduous broad-leaf forest)	0.006	15	0.002	14	0.001	15	<0.001	15
EBF (evergreen broad-leaf forest)	0.002	16	0.001	16	<0.001	19	<0.001	18
Cropland	0.002	16	0.001	16	<0.001	18		-
Paddy field	0.001	18	0.001	16	<0.001	16	<0.001	16
Grassland	0.001	18	0.001	16	<0.001	17	<0.001	17
DNF (deciduous needle-leaf forest)		-		-	<0.001	20	<0.001	19
Water bodies		-		-	<0.001	21		-
Bareland		-		-		-	<0.001	20
Bamboo forest		-		-		-		-
No data		-		-		-		-
Solar panel		-		-		-		-
Unclassified		-		-		-		-

Color-code

- Climate variables
- Elevation
- Landcover variables
